# Re-purposing software for functional characterization of the microbiome

**DOI:** 10.1186/s40168-020-00971-1

**Published:** 2021-01-09

**Authors:** Laura-Jayne Gardiner, Niina Haiminen, Filippo Utro, Laxmi Parida, Ed Seabolt, Ritesh Krishna, James H. Kaufman

**Affiliations:** 1 IBM Research, The Hartree Centre, Warrington, WA4 4AD UK; 2 IBM T. J. Watson Research Center, Yorktown Heights, NY 10598 USA; 3 IBM Almaden Research Center, San Jose, CA 95120 USA

**Keywords:** Microbiome, Functional analysis, Sequencing read classification, Taxonomy

## Abstract

**Background:**

Widespread bioinformatic resource development generates a constantly evolving and abundant landscape of workflows and software. For analysis of the microbiome, workflows typically begin with taxonomic classification of the microorganisms that are present in a given environment. Additional investigation is then required to uncover the functionality of the microbial community, in order to characterize its currently or potentially active biological processes. Such functional analysis of metagenomic data can be computationally demanding for high-throughput sequencing experiments. Instead, we can directly compare sequencing reads to a functionally annotated database. However, since reads frequently match multiple sequences equally well, analyses benefit from a hierarchical annotation tree, e.g. for taxonomic classification where reads are assigned to the lowest taxonomic unit.

**Results:**

To facilitate functional microbiome analysis, we re-purpose well-known taxonomic classification tools to allow us to perform direct functional sequencing read classification with the added benefit of a functional hierarchy. To enable this, we develop and present a tree-shaped functional hierarchy representing the *molecular function* subset of the Gene Ontology annotation structure. We use this functional hierarchy to replace the standard phylogenetic taxonomy used by the classification tools and assign query sequences accurately to the lowest possible molecular function in the tree. We demonstrate this with simulated and experimental datasets, where we reveal new biological insights.

**Conclusions:**

We demonstrate that improved functional classification of metagenomic sequencing reads is possible by re-purposing a range of taxonomic classification tools that are already well-established, in conjunction with either protein or nucleotide reference databases. We leverage the advances in speed, accuracy and efficiency that have been made for taxonomic classification and translate these benefits for the rapid functional classification of microbiomes. While we focus on a specific set of commonly used methods, the functional annotation approach has broad applicability across other sequence classification tools. We hope that re-purposing becomes a routine consideration during bioinformatic resource development.

**Video abstract**

**Supplementary Information:**

The online version contains supplementary material available at 10.1186/s40168-020-00971-1.

## Background

Analysis of the microbiome involves determining the microorganisms that are present in a given environment, and their respective functions [1]. Such analysis reveals what organisms are there, what they are doing, and how (by what molecular mechanisms) they are doing it. To date, a large variety of microbiome analyses have been conducted considering both sample context and investigative purpose. Such analyses range from microbiome analysis for fields such as animal health, agriculture and environmental studies [2, 3], to those focusing on human samples such as skin, saliva, stool or blood, since variation in the human microbiome has been linked to health conditions and diseases [4]. Typically, both 16S rRNA gene and whole metagenome shotgun sequencing can be used to identify the microorganisms that are present in a sample, i.e. the community structure, while further investigation is required to derive the functional potential of the microbial community from the sequence data [5, 6]. Additionally, metatranscriptome sequencing can be used to examine active functions [7]. The current standard approach is to first taxonomically and then to functionally classify sequencing reads derived from organisms that are present within a microbiome [8]. This approach for functional analysis of the microbiome can be laborious and computationally demanding.

Software such as Kaiju, Kraken 2, MEGAN, DIAMOND and HUMAnn2 firstly classify or align sequencing reads using a database of protein sequences, often with default databases such as the NCBI RefSeq or the microbial subset of the NCBI BLAST non-redundant protein database (nr) [9–14]. Some of the tools additionally utilize a reference taxonomy in this initial alignment or classification; however, they were largely designed for taxonomic profiling, where functional classification could follow, rather than direct functional profiling of the microbiome. Bahram et al. [15] reported the direct alignment of metagenomic reads to functional databases such as the Kyoto Encyclopedia of Genes and Genomes (KEGG) [16] using DIAMOND and the subsequent calculation of SEED functional module abundances [17]. KEGG terms for a gene or sequence directly indicate the function, process or component that the gene is involved in. Similarly, mi-faser [18] aligns microbiome sequencing reads using DIAMOND, to a reference database of microbial proteins with experimentally annotated molecular functions from KEGG. However, these methods do not directly associate reads with a functional hierarchical level or sub-level such as those implemented for taxonomic assignment of reads to their lowest common ancestor (LCA). Huson et al. took a step towards this goal with MEGAN using read alignment, e.g. DIAMOND, followed by an additional step to perform Gene Ontology-based (GO) functional classification. Their functional classification used a hierarchy with two sub-levels of GO terms below the domain root, e.g. molecular function. The first sub-level, with 84 nodes to refine GO-based classification and the second representing all GO-assigned InterPro families [19]. In contrast, we recently introduced PRROMenade [20, 21] for direct or one-step functional characterization of microbiomes, using a protein reference database and a four-level annotation tree derived from KEGG, supporting rapid functional rather than phylogenetic classification. With PRROMenade we proposed a novel labelling step that assigns query sequences directly to the lowest possible molecular function in the functional annotation hierarchy or tree. In this report, we propose that by generating a functional hierarchical structure, direct functional sequencing read classification is also possible by re-purposing current widely used tools. We use a deeper (10 level) GO-based hierarchy that allows more informative lowest common function binning of reads. Furthermore, we demonstrate that it is possible to utilize not only variable size sequence matching tools like PRROMenade and Kaiju, but also *k*-mer based methods (e.g. Kraken, Kraken 2) for rapid microbiome functional profiling. These methods avoid the computationally expensive full sequence alignment approach that has been the backbone of previous methods (e.g. MEGAN, mi-faser, HUMAnn2). We present our case with simulated as well as experimental datasets to demonstrate the feasibility of re-purposing commonly used taxonomic sequence classification tools for microbiome functional annotation.

Accurate functional classification requires a large-scale reference database of proteins annotated with functional labels, e.g. GO or KEGG terms. Therefore, software to perform such a task needs to be able to efficiently search this database while retaining sensitivity and specificity in matching sequence to the reference. Kraken and Kaiju are two well-known tools in the metagenomics community for taxonomic analysis. Kraken [11] is known for its robustness and speed [22], which is reflected in its high access rate (> 55,000 accesses) and citation rate (> 850 citations) since its introduction in 2014. Kraken classifies DNA sequencing reads using a DNA reference database. It assigns taxonomic labels to short DNA sequencing reads by examining the *k*-mers within each read and querying a database for those *k*-mers, for a fixed value of *k*. The Kraken reference database contains a mapping of every *k*-mer in the user reference genomic library to the LCA in a taxonomic tree of all genomes that contain that *k*-mer. The set of LCA taxa that correspond to the *k*-mers in a read are then analysed to create a best matching taxonomic label for the read [11]. The recently released Kraken 2 now allows classification of DNA reads using a protein reference and uses a compact hash table for its reference database allowing faster queries and lower memory requirements than its predecessor Kraken [13]. By comparison, Kaiju classifies DNA reads using a protein reference database allowing searching for variable length exact matches in amino acid databases with comparable speed and accuracy than methods using fixed size *k*-mers [9]. Kaiju also allows assignment of reads to an LCA and is fast becoming a popular tool in the metagenomic community. In this study, we demonstrate the applicability of these widely used tools for a new task, the task of functional classification with a functional labelling hierarchy.

PRROMenade was previously applied with a bacterial functional protein database from the IBM Functional Genomics Platform (IFGP) (formerly known as OMXWare) [23] and a functional labelling hierarchy based on KEGG to classify DNA sequencing reads. However, here we demonstrate that the same direct functional sequencing read classification is possible firstly, by re-purposing other widely used rapid read classification tools and secondly, by directly comparing DNA sequencing reads to a DNA reference rather than a protein database. This opens the door to allow a large range of popular existing taxonomic classification tools to be re-purposed for functional annotation. To extend previous work utilizing the KEGG functional labelling hierarchy, we derive a functional hierarchy based on the widely used Gene Ontology (GO) term molecular function database [24]. The IFGP database contains 40.1 million bacterial protein domains associated with 1740 GO molecular function codes and is therefore more than three times larger than the previously utilized subset of 11.9 million domains associated with KEGG Enzyme Nomenclature codes (EC). The GO term hierarchy is organized as a directed acyclic graph and therefore not directly amenable to conversion into a rooted functional hierarchy tree. We perform the necessary transformation of the graph into a rooted tree and adapt the resulting functional hierarchy to replace the phylogenetic taxonomy used by Kraken, Kraken 2 and Kaiju. We thus assign query sequences to the lowest possible node or molecular function in the functional annotation tree, i.e. the lowest common function that we denote here as LCF. This is in contrast to a standard phylogenetic taxonomic classification where sequences are assigned to their lowest common ancestor (LCA). We re-purpose a specific set of taxonomic classification tools based on their efficiency and wide-spread use, providing the necessary resources (reference database and taxonomy) to ensure that minimal effort on the behalf of the user is needed to apply their existing software’s to perform a new task. However, the proposed re-purposing approach has wide-ranging applicability for other metagenomic sequence classification tools and could pave the way for a new class of default reference databases and taxonomies for functional sequence classification. In the broader context, we demonstrate the feasibility of re-purposing rather than building from scratch during bioinformatic resource development.

## Results and discussion

### Transforming the GO hierarchy into a functional hierarchy tree

**Fig. 1:**
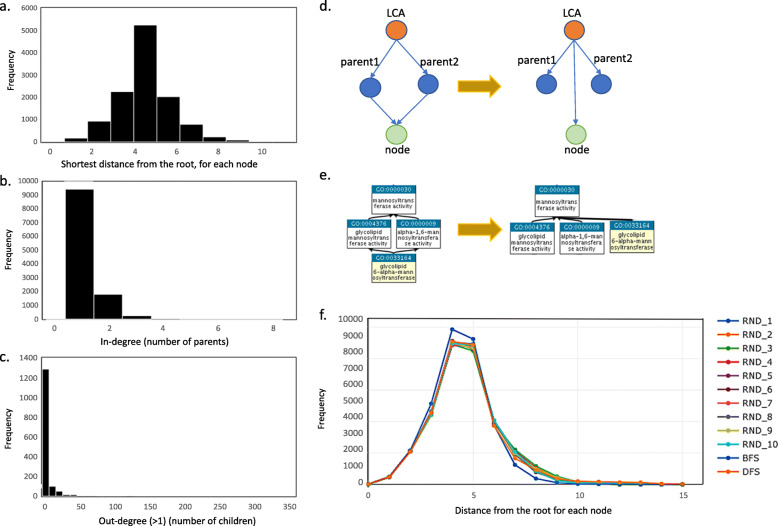
Transformation of GO hierarchy into a reference functional hierarchy tree structure. Here, GO terms are represented as nodes. **a** Histogram to show the frequency of the distance of each node from the root LCF of the entire molecular function ontology domain. **b** Histogram to show the frequency of the number of parents for each node also known as in-degree. **c** Histogram to show the frequency of the number of children or child terms directly associated with each node also known as out-degree if this number is > 1. Most nodes have only one parent. In cases where a node has 2 parent terms (second most common scenario), we show the most frequently encountered situation and our resolution strategy for this using a pictorial representation (**d**) and an actual example (**e**). **f** Showing the distance from each node to the root from our exploration of different tree building methods including DFS (depth-first search), BFS (breadth-first search) and in the cases where we have more than two parents and random single parents are selected (we repeated this 10 times, RND_1-RND_10)

The Gene Ontology (GO) hierarchy contains 11,684 terms in full. Although it has a pre-defined root, it is not a tree structure and therefore not directly amenable to conversion into a functional hierarchy. However, we were able to derive a rooted tree structure that includes nodes representing the GO molecular function terms in a depth-first search (DFS) order using the top-level term (GO:0003674) as the root LCF (see the “Methods” section). Such an approach could effectively maintain and maximize the representation of the topological order of the GO terms. We included all 11,684 terms and observed 10 levels in our GO-term-based functional annotation tree, with the majority of GO terms having a distance of 3–5 levels from the root node highlighting the resolution of the tree (Fig. 1a). The previous GO-term-based tree, developed by Huson et al. [19], encompassed a distance of only 2 levels from the same root node. This deep hierarchy allows a higher likelihood of more informative LCF (lowest common function) binning of reads. The vast majority of GO terms (that we treat as nodes in the hierarchy) were directly associated with a single parental GO term; however, we did note occurrences where GO terms had multiple parent terms (Fig. 1b, c). The second most common scenario was for a GO term to be directly associated with two parental GO terms or nodes (~ 1820 cases). These were resolved by connecting the child GO term to the single parent of the two parents, when possible. Figure 1d, e illustrates this situation and the resolution. In the few cases with more than two parents, one was randomly selected. We chose the depth-first search (DFS) approach for the tree structure derivation (see the “Methods” section) as it maximized the average distance of nodes from the root compared to breadth-first search (BFS) or random approach (RND), thus enabling more specific annotation of sequences (Fig. 1f).

### Functional classification of simulated reads using the GO hierarchy

The read simulation methodology (see the “Methods” section) was followed for sequences from the *Bordetella* genus of the phylum *Proteobacteria*, the *Salmonella* genus and the complete IFGP GO database. We used a range of substitution rates denoted as low (5%), medium (10%) and high (20%) substitution rates compared to the reference database, repeating our sequencing read simulation to represent organisms with a progressively less related (more divergent) sequence to the reference. Our aim was to mimic the natural variation that will be observed when comparing experimental sequencing datasets to these reference databases (see the “Methods” section).

We used *Bordetella*, *Salmonella*, and the complete IFGP database of sequences to assess the impact of different sized sets of GO terms in the reference database on the ability to discriminate between them, using reads with low substitution rates (5% divergent from the reference sequences). Using Kraken [11] with the three custom databases to classify the corresponding three sets of simulated DNA sequencing reads, we could classify 84.07% of the reads for *Bordetella*, 84.63% of the reads for *Salmonella* and 85.2% of the reads for the full IFGP database (Supplementary Table S1). Furthermore, 77.59% of the reads for *Bordetella*, 34.64% of the reads for *Salmonella* and 18.9% of the reads for IFGP database were classified to the exact same GO term that the read originated from. Otherwise, 92.46%, 61.45% and 84.10% of *Bordetella*, *Salmonella* and IFGP full database sequences, respectively, were classified as either the exact same GO term that the read originated from or as a term that was functionally related to it in the hierarchy that was not the root LCF (see the “Methods” section). Increasing the size of the GO-term-based database may reduce the number of sequences assigned to the exact GO term of origin but it does not affect the ability to assign the sequences to a suitable functionally related term further up in the hierarchy. Assessing those reads that were associated with an incorrect or un-related GO term gives us an error rate of < 0.01% for *Bordetella*, 0.95% for *Salmonella* and 1.7% for the full IFGP database.

We noted (Supplementary Table S1) that 7.53%, 37.60% and 14.20% of the *Bordetella*, *Salmonella* and full IFGP database reads that were classified as a specific GO term were assigned to GO:0003674, which although a specific term, represents the entire ontology domain of molecular function and is in effect the root or LCF of this GO domain. *Salmonella* appears to be particularly problematic with regard to assigning reads to the root rather than a more specific functionally related term. We hypothesized that the result for *Salmonella* potentially reflects the public interest in sequencing *Salmonella enterica* isolates; hence, the database includes several closely related strains leading to increased ambiguity for reads from non-variable parts of the genome. In support of this hypothesis, collapsing identical sequences from the *Salmonella* reference database (leaving the longest representative) dramatically improved root classification with only 7.46% of reads aligning to the root LCF which is more comparable to the 7.53% observed for *Bordetella*. Notably, the root bias for read classification is not observed as predominantly for the complete IFGP database that has undergone large scale redundancy removal to effectively incorporate many genera including *Salmonella*. In fact, aligning the simulated *Salmonella* derived reads to the full IFGP database, the problem also largely resolves, where we observe 12.5% of reads aligning to the root LCF.

The results that were obtained using Kraken for classification of simulated reads (low substitution rate) to the complete IFGP database were compared to those derived from Kaiju, Kraken 2 and PRROMenade classification (Table 1). We consider Kaiju with the MEM (maximal exact matching) option and PRROMenade together since they have the same method of classification and therefore produce the same summary statistics, only the running time varies, where PRROMenade performs classification in less than half the time of Kaiju [20]. Therefore, we refer to Kaiju and PRROMenade collectively as “MEM approaches” in the text. Comparing Kraken, Kraken 2 and the MEM approaches, we also ascertain the impact of using DNA-DNA compared to using a DNA-protein comparison for functional classification. MEM approaches are able to classify a similar proportion of the simulated (low substitution rate) sequencing reads to a protein database (85.1%) compared to the 85.2% classified by Kraken using a DNA database. This is perhaps surprising since proteins are more conserved than the underlying DNA. Therefore, protein sequence comparison can be more tolerant to sequencing errors and distant evolutionary matches due to the degeneracy of the genetic code. As such, protein databases are implemented typically for functional sequence analysis in metagenomics and are expected to allow more divergent sequencing reads to be classified. The comparable proportion of reads classified using a DNA-based database may reflect our use of sequencing reads that are not highly divergent from the reference database. Kraken 2 showed improved performance compared to both the MEM approaches and to Kraken, classifying 93.91% of the reads. If one focuses on the classified reads and the ability of the software to classify them correctly to a (non-root) LCF, Kraken, Kraken 2 and the MEM approaches are highly comparable, classifying 84.1%, 86.3% and 84.7% of reads, respectively. Kraken, Kraken 2 and the MEM approaches also show a consistently low error rate in general at 1.7%, 1.12% and 0.68%. Finally, we noted that ~ 48% of reads matched GO terms at a distance of 5 or more levels from the root highlighting the utility of the resolution of our hierarchy.

**Table 1:** Functional classification of 474,930,848 simulated (low substitution rate) sequencing reads by Kraken, Kraken 2 and MEM approaches (Kaiju and PRROMenade) using the full IFGP sequence database

Software	Database format	% Unclassified reads	% Classified Reads	% Classified reads assigned to the root	% Classified reads assigned to correct GO term or related non-root LCF	% Classified reads assigned to an incorrect GO term
Kraken	DNA	14.80	85.20	14.20	84.10	1.70
Kraken 2	Amino Acid	6.09	93.91	13.71	86.29	1.12
MEM	Amino Acid	14.86	85.14	14.61	84.71	0.68

Above, we compared the results of Kraken to those derived from Kraken 2 and MEM approaches for simulated sequencing reads with high similarity (low substitution rate) to the reference database, classified using the complete IFGP database. Next, we investigate the effect on the classification of using sequencing reads that diverge more strongly from the reference database. Firstly, we do this using simulated reads since we know which functional groups the sequencing reads were derived from enabling determination of the accuracy of the classifications. We use simulated reads that have medium (10%) and high (20%) substitution rates compared to their derived reference. Secondly, we apply the methods on soil metagenome samples for comparison of classification rates on experimental data.

**Fig. 2:**
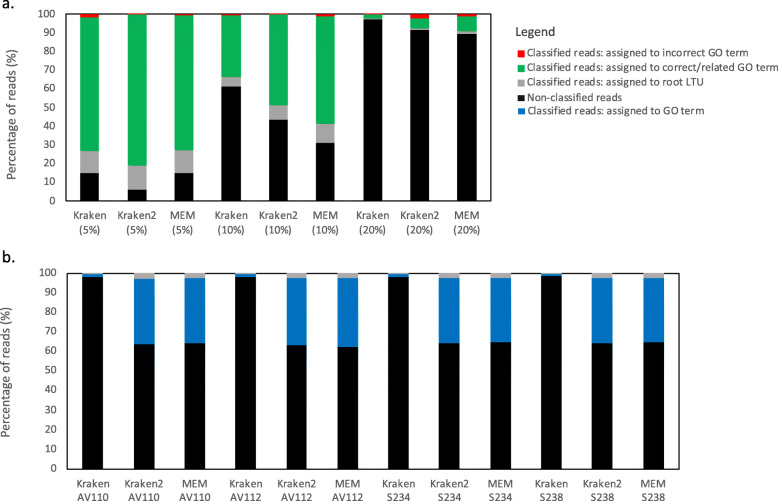
Analysis of the classified reads by Kraken, Kraken 2 and MEM approaches (Kaiju and PRROMenade). **a** Stacked bar chart to show the percentage of reads that were classified and non-classified using the complete IFGP database by Kraken, Kraken 2 and MEM approaches using simulated reads with 5, 10 and 20% substitution rates. The classified reads are divided into categories to show the proportions of the classified reads that were assigned to either the root, an incorrect GO term or finally to a correct or related GO term that was not the root LCF. **b** Stacked bar chart to show the percentage of reads that were classified and unclassified using the complete IFGP database by Kraken, Kraken 2 and MEM approaches using four experimental samples AV110 and AV112 (moist tropical forest) and S234 and S238 (boreal forest). The classified reads are divided into categories to show the proportions of the classified reads that were assigned to a GO term or the root

It is clear from Supplementary Table S2 and Fig. 2 that across all the tested software, increasing substitution rate in our simulated reads decreases the number of classified reads while also increasing the number of reads that are assigned to an incorrect GO term (Fig. 2a). The proportion of classified reads assigned to the root is largely conserved across the tools and read substitution rates. With a 5% substitution rate in the simulated reads, all tools perform comparably. However, when this substitution rate increases to 10% there is more variation between the tools with the MEM approaches classifying the most reads (68.8%), Kraken 2 slightly lower at 56.3% and Kraken falling behind classifying only 38.8% as its DNA reference matching potentially hinders the ability to assign more divergent sequences compared to the protein reference matching methods. Finally, using a 20% substitution rate in the simulated reads all methods perform relatively poorly with the percentage of classified reads ranging from 2.5 to 10.2%.

### Functional classification of the soil microbiome using the GO hierarchy

We used experimental metagenomic samples for functional classification. We selected four soil metagenomic samples from the study by Bahram et al. [15] since soil represents one of the most diverse microbiomes on earth and as such one of the most difficult to characterize. The four samples represented habitats ranging from Boreal forests to Moist tropical forests. The MEM approaches were able to classify 35.93% of the sequencing reads on average, comparable to 36.17% by Kraken 2, but Kraken only classified 1.65% (Fig. 2b, Supplementary Table S3). This, alongside our previous analysis, fits our hypothesis that protein databases will allow more divergent sequencing reads to be classified where a DNA to DNA classification may fail. From a performance perspective, compared to our simulated read analysis and using Kraken 2 and the MEM approaches as a guideline, the soil metagenomic reads reflect the classification statistics that we may expect from simulated reads with a ~ 15% substitution rate (Supplementary Figure S1). When we focus on those reads that can be informatively assigned to a GO term below the root, this represents 88.81%, 93.16% and 93.28% or the vast majority of classified reads for Kraken, Kraken 2 and the MEM approaches respectively (Fig. 2c).

We compared our best LCF classification approach for the soil datasets (Kraken 2) with the sequence-similarity search approach (alignment) that was taken in the original study [15] (DIAMOND [10], percentage identity 50% and *e* < 1 × 10^−9^), applying both with the IFGP database. Here, we compare using our best LCF classification as an example, although results with Kraken 2 and the MEM approaches were largely indistinguishable. The results showed that our best LCF classification approach, which classified 36.17% of reads on average, classifies a higher proportion of reads than the average of 23.0% aligned reads that we observe using DIAMOND. Furthermore, this is a more than 4-fold increase upon the 7.8% of reads aligned using the same software and parameters from the original study combined with the KEGG database [16] that was reportedly used. To highlight the insight that our functional annotation method could provide, using Kraken 2 and the IFGP database, we analysed the 189 soil metagenomic samples from the study by Bahram et al. [15] to enable comparison (see the “Methods” section). Our approach provides additional insights to those that were presented in the original study allowing us to highlight the enrichment in specific functional groups across different latitudes compared to other functional groups. We noted that the functional group GO:0016209 related to antioxidant activity (Supplementary Figure S2a) showed a correlation with latitude (*R*^2^ = 0.2186). GO:0016209 showed a significant decrease in the proportion of reads associated with antioxidant activity in equatorial samples (latitude between − 20 and 20) compared to polar samples were antioxidant activity was enriched (latitude less than − 40 or greater than 40) (*P* < 0.001, *t* = 5.6774, df = 127). This is supported by observations that soil antioxidant capacity positively correlates with soil carbon [25] and that soil carbon is depleted in equatorial regions compared to higher latitudes due to metabolic activity and species richness of soil organisms generally increasing toward equatorial regions [26]. Furthermore, we noted that the functional group GO:0045735 related to nutrient reservoir activity (Supplementary Figure S2b) showed weak correlation with latitude (*R*^2^ = 0.1235) where this functional profile shows no difference between the equator and the poles, however, becomes significantly depleted in mid-latitudes (latitude from − 40 to − 20 or 20 to 40) compared to both the equatorial (*P* = 0.0026, *t* = 3.0727, df = 117) and the polar regions (*P* = 0.0035, *t* = 2.9837, df = 114).

### Comparison with commonly used functional annotation approaches

Several methods exist for functional profiling of metagenomes [19, 27–30]. These methods typically involve sequence-similarity searching (or alignment) rather than classification. Many of these methods benefit from the recent methodological advances in translated search and they typically interpret the translated taxonomic search of sequencing reads to assign metabolic functions to them downstream [10, 31]. This means that such methods are often less straight-forward and slower compared to our direct functional classification; however, they can profile functional content at a high-level resolution to understand the roles of specific genes in a given context, whereas our method provides a community-level profile of functional content. Focusing on some of the most highly cited methods, we compare functional annotation alignment approaches to our use of sequence classification software to assign the LCF to a read. Firstly, MEGAN uses read alignment to a protein database e.g. using sensitive BLAST [32] alignment or employing a faster but less sensitive heuristic method, DIAMOND [10], to achieve acceptable performance. Followed by an additional step to perform functional classification [19]. Similarly, mi-faser [18] aligns metagenomic sequencing reads to bacterial protein sequences with annotated molecular functions from KEGG using DIAMOND [10]. The third functional annotation approach, HUMAnN2 (HUMAnN’s successor) [12], is another alignment-based method for functional characterization. It first attempts to identify the most abundant species in the sample and maps the reads with Bowtie 2 [33] against their pangenome. In subsequent steps, a DIAMOND [10] search can also be performed on unaligned reads and alignments are interpreted to assign functional information.

Although we develop a functional hierarchy and re-purpose taxonomic sequence classification software for functional annotation, our GO term reference database can still be used with other alignment software for functional annotation. Therefore, we compared mapping of our simulated (low substitution rate) reads to the complete IFGP database using Bowtie 2 and DIAMOND (“alignment tools”) to mapping with Kraken, Kraken 2 and our MEM approaches (“classification tools”). The classification tools give a significant speed advantage with some also having significantly lower memory requirements (Table 2). Our fastest classification (Kraken 2) provides an 80-fold speed increase to the fastest alignment tool. Unlike our classification tools, the alignment tools do not integrate a taxonomic or functional hierarchy to assign reads to their LCA or LCF. As such, when we focus on those reads assigned to multiple locations with equal likelihood, where the LCA/LCF approach makes an informed decision on the read’s “best” location, approaches such as Bowtie 2 typically report a random best location which is less informative. This was a common problem with 87.1% of aligned reads assigned to multiple reference locations with the same quality score by Bowtie 2. Therefore, although there is a small decrease on average in classified/aligned reads from the classification tools compared to alignment tools (except for Kraken 2 which outperforms Bowtie 2), these reads may not be as informatively annotated from a functional perspective.

**Table 2:** Classification of 474,930,848 simulated sequencing reads (low substitution rate) by Kraken, Kraken2, the MEM approaches Kaiju and PRROMenade compared to alignment using Bowtie2 and DIAMOND. Analysis conducted using the full IFGP GO term sequence database and functional annotation

Software	Speed (user time + system time in seconds)	Peak memory (GB)	% Classified/aligned reads	Parameters
Diamond (MEGAN/mi-faser/HUMAnN2)	9,791,017	21.61	98.82	-k 1
Bowtie2 (HUMAnN2)	758,766	58.26	92.95	-S (output SAM file)
Kaiju	456,225	16.44	85.14	-a mem
PRROMenade	219,483	140.38	85.14	taxonomic-classifierMem
Kraken	23,220	35.2	85.20	Defaults
Kraken 2	9466	1.95	93.91	Defaults

## Conclusions

We report direct functional microbiome read classification by re-purposing commonly used taxonomic classification tools and demonstrate this using both protein and DNA sequence databases. Moreover, we do this by developing and using a functional hierarchy that overcomes problems encountered by reads matching multiple sequences equally well (evidenced by our analysis using Bowtie 2), by assigning reads to their LCF. For less divergent sequence sets, such as our simulated read set with a substitution rate of 5%, we can assign the majority of sequencing reads to an informative (non-root) and correct LCF using any of the methods (demonstrated using popular *k*-mer based tools Kraken, Kraken 2 and MEM approaches including PRROMenade and Kaiju). We observe a similar overall read classification rate and accuracy for both protein and DNA reference databases. The error rate for sequence classification is also low at 0.68–1.7% in the simulated setting. Protein sequence databases are frequently utilized for functional sequence analysis in metagenomics since they allow diverse sequencing reads to be classified, when the respective amino acids match. We also test classification using simulated reads with a high substitution rate and using experimental soil metagenomic samples where we reveal new biological insights. We observe a characteristic increase in the number of classified reads using the protein database compared to a DNA database when these diverse samples are analysed. However, all methods assign similar proportions of their classified reads informatively to a GO term or an ancestral/related (non-root) LCF GO term. Interestingly, when the substitution rate of our simulated reads is low (5%), Kraken 2 marginally classifies the most reads; however, when the substitution rate increases to 10%, the MEM approaches significantly outperform it classifying the most reads (68.8% compared to 56.3%).

We focus on functional read classification by re-purposing Kraken, Kraken 2 and Kaiju due to their prevalent usage in the community; however, this methodology has broad applicability across microbiome sequence classification tools. While we focus here on GO terms relating to molecular function, this analysis could be extended to include additional GO terms for biological processes and cellular components, or any other coding system that can be represented or adapted to a tree of hierarchical annotations. Furthermore, our bacterial focused functional reference database could be extended to include other microbes or exchanged to primarily focus on other microbes. Ultimately, we hope that re-purposing becomes a consideration more generally, where developers support and diversify reference databases routinely for bioinformatic resources to allow adaptation of their tools for new purposes. Such software diversification could be identified by the developers themselves incorporating additional searches during routine updates or led by their user-base if feedback opportunities or user requests are enabled and encouraged. We propose that a good candidate software for re-purposing, would be typically highly used, well-documented, well-maintained and optimized. As we highlight here, re-purposing ideas have the potential to introduce a new biological concept for a software while also offering an alternative solution to a computationally inefficient task.

## Methods

### Transforming the GO hierarchy into a functional hierarchy tree

Our derived tree structure includes nodes that are representative of the GO molecular function subset. To adapt the GO hierarchy into a tree structure, we first transformed it into a rooted tree by visiting the nodes in depth-first search (DFS) order, starting from the top-level code for molecular function as the root LCF. We also explored breadth-first search (BFS) order and random removal of edges for multi-parent nodes. However, the DFS approach maximized the average distance of nodes from the root, thus enabling more specific annotation of sequences (Fig. 1e). Finally, on occasions where multiple GO terms were associated with a single protein sequence in the reference database, we selected a representative GO term prioritizing the most specific term that was available.

### Development of reference databases and taxonomy

For this analysis, we used a large-scale database called the IBM Functional Genomics Platform, IFGP [23]. At the time of this study, IFGP contained 138 million bacterial protein domains. A subset of 11.9 million domains, totalling 3.7 billion amino acids, had associated KEGG Enzyme Nomenclature codes (EC). In addition, the IFGP database contained Gene Ontology (GO) terms [24] for 40.1 million domains totalling 9.8 billion amino acids and associated with 1740 GO molecular function codes. In order to test the functional read classification, we developed three DNA sequence databases and three associated functional hierarchies. These three test sets were derived from the IFGP database with increasing sizes; the first a relatively small test set of sequences from the *Bordetella* genus of the phylum *Proteobacteria*, the second a larger sequence set (50× larger) from the *Salmonella* genus of the family *Enterobacteriaceae*, and finally the complete IFGP GO database of sequences [24]. The analysis using databases of increasing size allows us to ascertain if a larger set of GO terms in our reference database affects the ability to discriminate between them and therefore affect sequence classification precision and accuracy.

Firstly, for *Bordetella*, 40,127 protein sequences were labelled with 689 GO terms. Each GO term had an average of 199.4 sequences associated with it (89.3% of GO terms had > 10 sequences associated with them). Secondly, for *Salmonella*, 4,431,494 sequences were labelled with 1222 GO terms. Finally, the complete IFGP GO database contained 40,509,561 sequences that were labelled with 1740 GO terms. For use with Kraken, all of these protein sequences were reverse translated to DNA sequences using EMBOSS backtranseq [34].

A *k*-mer database was constructed using the software Kraken (v0.10.5) [11] for the above three databases (default settings). Kraken assigns taxonomic labels to short DNA reads based on the LCA most closely matching its *k*-mer profile. Here, we applied Kraken in a different way; we created three custom *k*-mer databases that contained our GO reference DNA sequence sets and a custom “taxonomy” (Additional file 2-nodes.dmp and Additional file 3-names.dmp) to include nodes and their relationships that are representative of the GO terms’ functional hierarchical structure. This allows Kraken to assign GO terms, rather than its typical taxonomic classification, to short DNA reads. Here, we focused on molecular function GO terms and as such our root LCF in the functional hierarchy was the GO term GO:0003674 that corresponds to the term “molecular function”. Note that reference databases in Kraken (as developed here) are also compatible for downstream usage with KrakenUniq [35].

For comparative analyses with Kraken 2, Kaiju and PRROMenade, we developed reference databases from the complete IFGP database. However, for use with Kraken 2, Kaiju and PRROMenade the IFGP protein sequences sets could be used directly for database generation with no need for DNA conversion. The corresponding custom GO term functional hierarchy (Additional file 2-nodes.dmp and Additional file 3-names.dmp) was used. Kraken, Kraken 2, PRROMenade and Kaiju were run with default settings except for the choice of the mem scoring function for Kaiju since this results in a closer comparability of the alignment methodologies used. PRROMenade is theoretically equivalent to the Kaiju with the “MEM” option selected, while being faster in practice to run [20].

### Simulated sequence data

To demonstrate our ability to discriminate between the GO reference sequences in our custom databases, we used Kraken, Kraken 2, PRROMenade and Kaiju to assign GO terms to simulated sequencing datasets that were generated from the databases themselves. Sequencing reads were simulated randomly from sequences in each of the three reference databases *Bordetella*, *Salmonella* and the complete IFGP database. The original protein sequences from the respective databases were reverse translated to DNA sequences using EMBOSS backtranseq [34] and then sequencing reads were simulated from these DNA sequences using SAMtools WGSIM (v. 0.3.1-r13) [36]. We generated paired-end sequencing reads of 125 bp in length. To avoid identical sequencing reads to the reference we used a substitution rate of 0.05 (5%) between the reference and the sequencing reads—these reads represent the most similar reads to the reference that we use in this study (95% similar) and we refer to these reads as our “low substitution rate” reads. We also generated paired-end sequencing reads of 125 bp in length with higher substitution rates of 0.10 (10%) and 0.20 (20%) to allow comparison and to represent less and less related organisms with a more divergent sequence—we refer to these reads as “medium substitution rate” and “high substitution rate”, respectively.

When the read simulation methodology was followed for sequences from the *Bordetella* genus of the phylum *Proteobacteria*, we generated 665,699 read pairs resulting in ~ 10× coverage of the reference sequences and inclusion of 96.72% of GO sequences that were of sufficient length (125 bp or longer) to generate sequencing reads from. Secondly, for sequences from the *Salmonella* genus, we generated 175,635,320 read pairs resulting in ~ 10× coverage of the reference sequences and inclusion of 96.78% of GO sequences. This methodology was followed for the complete IFGP GO database where we generated 237,465,424 read pairs resulting in ~ 2× coverage of the reference sequences and inclusion of 89.2% of GO sequences (9.8% of the sequences were skipped due to having a length shorter than 125 bp, while 1% were skipped because they contained a high proportion of ambiguous bases or Ns).

### Evaluation criteria for classification of sequencing reads

The definitions used in this analysis identify unclassified reads as reads having no match to the reference database (although these reads may be automatically denoted as root by some methods, we only include classified reads in our calculations of reads assigned to the root). Classified reads are those that were matched to a sequence in the reference database. For reads that we simulated from sequences, we encode in the read ID the specific GO term that the sequence of origin was associated with, this allows us to compare the GO term classification of the read to its GO term of origin. This comparison can result in firstly, an exact match between the GO term that a read is classified to compared to its origin. Secondly, no exact match but a match between the GO term that a read is classified to compared to a term that is higher up in the GO functional hierarchy but on the same path as the origin GO term. We use the GO functional hierarchy paths to derive such matches and refer to them as correct matches to non-root, related matches or ancestral matches (Additional file 4). Thirdly, a classified read can be assigned to the root LCF GO term. Finally, if neither of the previous three matching options are observed, a read is determined to have been assigned to an incorrect GO term.

### Analysis of experimental soil dataset

For 189 soil metagenome paired-end read sequencing samples from the study by Bahram et al. [15], Kraken 2 was run with default settings for each sample using the complete IFGP database as a reference database alongside the corresponding custom GO term functional hierarchy (Additional file 2-nodes.dmp and Additional file 3-names.dmp). Each aligned read pair (DIAMOND) was assigned to a single location and each classified read pair (Kraken 2) was assigned to a single functional hierarchy node. Classified reads (Kraken 2) were summed for each of the following selection of level 2 GO terms (summed across those related GO terms that were lower in the functional tree hierarchy): binding [GO:0005488], structural molecule activity [GO:0005198], catalytic activity [GO:0003824], [GO:0004871], cargo receptor activity [GO:0038024], antioxidant activity [GO:0016209], molecular carrier activity [GO:0140104], transporter activity [GO:0005215], translation regulator activity [GO:0045182], transcription regulator activity [GO:0140110], molecular function regulator [GO:0098772], hijacked molecular function [GO:0104005], nutrient reservoir activity [GO:0045735], protein tag [GO:0031386] and toxin activity [GO:0090729]. Read numbers were normalized to a scale of 0–1 for each sample to allow comparison. Scatterplots were drawn to show the proportion of aligned reads to each GO term group for each of the 189 samples after alignment (Supplemental Figure S2). The respective trendlines of closest fit for the datapoints are shown in the figure (in red) and were determined according to *R*^2^ scores: for GO:0016209 y = 1E−14x^6^−2E−13x^5^−3E−11x^4^−6E−10x^3^+7E−08x^2^+7E−07x+0.0014 and *R*^2^ = 0.2186, for GO:0045735 y = 3E−11x^4^−4E−10x^3^−8E−08x^2^−3E−07x+0.0004 and *R*^2^ = 0.1235, for GO:0005488 y = 8E−10x^4^−2E−08x^3^−2E−06x^2^−6E−06x+0.2742 and *R*^2^ = 0.0175, for GO:0098772 y = 3E−15x^6^+3E−13x^5^+2E−11x^4^−2E−09x^3^−3E−08x^2^+1E−06x+0.0008 and *R*^2^ = 0.0892, for GO:0003824 y = − 2E−09x^4^−5E−10x^3^+4E−06x^2^+0.0001x+0.6043 and *R*^2^ = 0.0532, for GO:0005215 y = 6E−10x^4^−4E−09x^3^−1E−06x^2^−4E−05x+0.085 and *R*^2^ = 0.062 and for GO:00140110 y = 5E−06x+0.0194 and *R*^2^ = 0.007.

## Supplementary Information

**Additional file 1: Figure S1.** Estimation of substitution rate of soil metagenomic samples. Scatterplot to show the classification rate (y-axis) of Kraken2 (pink datapoints) and the MEM approaches Kaiju and PRROMenade (blue datapoints) for simulated reads as substitution rate increases (x-axis)**.** The linear trendline for the datapoints is shown in black with its respective linear equation. The red line denotes the average classification rate of the soil metagenomic samples considering both Kraken2, Kaiju and PRROMenade to estimate the substitution rate of these samples if we were to attempt to recreate them from simulated reads. **Figure S2.** Read alignment of 189 soil metagenomic samples to GO functional groups. Scatterplots to show the proportion of classified reads to each GO term for each of the 189 analysed soil samples after classification to the IFGP GO term sequence database using Kraken2 (read numbers normalized to a scale of 0-1 for each sample to allow comparison). The respective trendlines of closest fit for the datapoints are shown in red. Here we show classification summarized for specific GO functional groups as follows; **(a)** GO:0016209 for antioxidant activity, **(b)** GO:0045735 for nutrient reservoir activity, **(c)** GO:0005488 for binding, **(d)** GO:0098772 for molecular function regulation, **(e)** GO:0005198 for structural molecule activity, **(f)** GO:0003824 for catalytic activity, **(g)** GO:0005215 for transporter activity and **(h)** GO:00140110 for transcription regulator activity. **Table S1.** Classification of simulated sequencing reads (low substitution rate) by Kraken using three different sized GO term sequence databases (DNA based) and functional annotation. **Table S2.** Classification of 474,930,848 simulated sequencing reads (low, medium and high substitution rate) by Kraken, Kraken2 and the MEM approaches Kaiju and PRROMenade using the full IFGP GO term sequence database and functional annotation. **Table S3.** Classification of real soil metagenome sequencing reads by Kraken, Kraken2 and the MEM approaches Kaiju and PRROMenade using the full IFGP GO term sequence database and functional annotation.

**Additional file 2.** nodes.dmp.

**Additional file 3.** names.dmp.

**Additional file 4.** GO reference functional hierarchy tree structure.

**Additional file 5.** A step by step description of how to use our GO functional hierarchy to build reference databases and perform classification for Kraken2 and Kaiju.

**Additional file 6.** Building a reference database using the GO taxonomy (Kaiju).

**Additional file 7.** Building a reference database using the GO taxonomy (Kraken2).

**Additional file 8.** Sample GO sequence database.

## Data Availability

The experimental soil metagenomics datasets that were used in this study (Bahram et al., 2018) are available from the NCBI Sequence Read Archive under project name PRJEB18701 at https://trace.ncbi.nlm.nih.gov/Traces/sra/?study=ERP020652. The IFGP Gene Ontology (GO) annotated database has previously been published (Seabolt et al., 2019). The simulated sequencing reads that were generated in this study are available from the European Nucleotide Archive repository (at https://www.ebi.ac.uk/ena), study PRJEB36971. The following data sources that were generated during this study are included in this published article and its supplementary information files: the functional hierarchy files nodes.dmp and names.dmp, used in Kraken/Kraken 2 for database construction, are available as Additional files 2 and 3, respectively (only the nodes.dmp file was used for Kaiju), and the GO reference functional hierarchy tree structure is available as Additional file 4 where each row represents a branch and followed from left to right progresses from the most specific term to the root. A step by step description of how to use our GO functional hierarchy to build reference databases and perform classification for Kraken2 and Kaiju is available as Additional file 5. Additional file 5 also describes the usage of Additional files 6, 7 and 8 that contain GO hierarchy files in formats specific to Kaiju and Kraken2 and sample database sequences.

## References

[1] Claesson MJ, Clooney AG, O’Toole PW (2017). A clinician’s guide to microbiome analysis. Nat Rev Gastroenterol Hepatol..

[2] Pace NR (1986). The analysis of natural microbial populations by ribosomal RNA sequences. Adv Microb Ecol.

[3] Venter JC (2004). Environmental genome shotgun sequencing of the Sargasso Sea. Science..

[4] Gilbert JA, Blaser MJ, Caporaso JG, Jansson JK, Lynch SV, Knight R (2018). Current understanding of the human microbiome. Nat Med..

[5] Janda JM, Abbott SL (2007). 16S rRNA gene sequencing for bacterial identification in the diagnostic laboratory: pluses, perils, and pitfalls. J Clin Microbiol..

[6] Jovel J, Patterson J, Wang W, Hotte N, O’Keefe S, Mitchel T, Perry T, Kao D, Mason AL, Madsen KL (2016). Characterization of the gut microbiome using 16S or shotgun metagenomics. Front Microbiol..

[7] Shakya M, Lo C, Chain PSG (2019). Advances and challenges in metatranscriptomic analysis. Front Genet.

[8] Langille MG (2018). Exploring linkages between taxonomic and functional profiles of the human microbiome. mSystems.

[9] Menzel P, Ng KL, Krogh A (2016). Fast and sensitive taxonomic classification for metagenomics with Kaiju. Nat Commun..

[10] Buchfink B, Xie C, Huson DH (2015). Fast and sensitive protein alignment using DIAMOND. Nat Methods..

[11] Wood DE, Salzberg SL (2014). Kraken: ultrafast metagenomic sequence classification using exact alignments. Genome Biol..

[12] Franzosa E, McIver L, Rahnavard G, Thompson L, Schirmer M (2018). Species-level functional profiling of metagenomes and metatranscriptomes. Nat Methods..

[13] Wood DE, Lu J, Langmead B (2019). Improved metagenomic analysis with kraken2. Genome Biol.

[14] Mitra S, Rupek P, Richter DC (2011). Functional analysis of metagenomes and metatranscriptomes using SEED and KEGG. BMC Bioinformatics.

[15] Bahram M, Hildebrand F, Forslund S, Anderson J, Soudzilovskaia N (2018). Structure and function of the global topsoil microbiome. Nature.

[16] Kanehisa M, Goto S (2000). KEGG: Kyoto Encyclopedia of Genes and Genomes. Nucleic Acids Res.

[17] Overbeek R, Begley T, Butler RM, Choudhuri JV, Chuang HY, Cohoon M (2005). The subsystems approach to genome annotation and its use in the project to annotate 1000 genomes. Nucleic Acids Res..

[18] Zhu C, Miller M, Marpaka S, Vaysberg P, Ruhlemann M (2018). Functional sequencing read annotation for high precision microbiome analysis. Nucleic Acids Res..

[19] Huson DH (2016). MEGAN Community Edition - Interactive Exploration and Analysis of Large-Scale Microbiome Sequencing Data. PLoS Comput Biol.

[20] Utro F, Haiminen N, Siragusa E, Gardiner LJ, Seabolt E, Krishna R, Kaufman JH, Parida L (2020). Hierarchically labeled database indexing allows scalable characterization of microbiomes. iScience.

[21] Haiminen N, Utro F, Seabolt E, Parida L. Functional pathways in respiratory tract microbiome separate COVID-19 from community-acquired pneumonia patients. bioRxiv. 2020; 10.1101/2020.05.01.073171.

[22] Tausch SH, Strauch B, Andrusch A, Loka TP, Lindner MS, Nitsche A, Renard BY (2018). LiveKraken – real-time metagenomic classification of illumina data. Bioinformatics..

[23] Seabolt EE, Nayar G, Krishnareddy H, Agarwal A, Beck KL, Terrizzano I, Kandogan E, Roth M, Mukherjee V, Kaufman JH (2019). IBM Functional Genomics Platform, a cloud-based platform for studying microbial life at scale. arXiv Preprint arXiv.

[24] Ashburner M, Ball CA, Blake JA, Botstein D, Butler H, Cherry JM, Davis AP, Dolinski K, Dwight SS, Eppig JT (2000). Gene Ontology: tool for the unification of biology. The Gene Ontology Consortium. Nat Genet.

[25] Rimmer DL (2009). Antioxidants in soil organic matter and in associated plant materials. Eur J Soil Sci.

[26] Madeira F, Park YM, Lee J (2019). The EMBL-EBI search and sequence analysis tools APIs in 2019. Nucleic Acids Res.

[27] Silva GG, Green KT, Dutilh BE, Edwards RA (2016). SUPER-FOCUS: a tool for agile functional analysis of shotgun metagenomic data. Bioinformatics..

[28] Sharma AK, Gupta A, Kumar S, Dhakan DB, Sharma VK (2015). Woods: a fast and accurate functional annotator and classifier of genomic and metagenomic sequences. Genomics.

[29] Petrenko P, Lobb B, Kurtz DA, Neufeld JD, Doxey AC (2015). MetAnnotate: function-specific taxonomic profiling and comparison of metagenomes. BMC Biol.

[30] Abubucker S (2012). Metabolic reconstruction for metagenomic data and its application to the human microbiome. PLoS Comput Biol.

[31] Zhao Y, Tang H, Ye Y (2012). RAPSearch2: a fast and memory-efficient protein similarity search tool for next-generation sequencing data. Bioinformatics..

[32] Boratyn GM (2013). BLAST: a more efficient report with usability improvements. Nucleic Acids Res..

[33] Langmead B, Salzberg S (2012). Fast gapped-read alignment with Bowtie 2. Nat Methods..

[34] Chojnacki S, Cowley A, Lee J, Foix A, Lopez R (2017). Programmatic access to bioinformatics tools from EMBL-EBI update: 2017. Nucleic Acids Res..

[35] Breitwieser FP, Baker DN, Salzberg SL (2018). KrakenUniq: confident and fast metagenomics classification using unique k-mer counts. Genome Biol..

[36] Li H, Handsaker B, Wysoker A, Fennell T, Ruan J (2009). The Sequence Alignment/Map format and SAMtools. Bioinformatics..

