# Supplementary material for: Re-purposing software for functional characterization of the microbiome
Source: Microbiome. 2021 Jan 9;9:4. doi: 10.1186/s40168-020-00971-1 (PMC7797099; doi:10.1186/s40168-020-00971-1)
Supplement: Supplementary file 2 — Additional file 1: Figure S1. Estimation of substitution rate of soil metagenomic samples. Scatterplot to show the classification rate (y-axis) of Kraken2 (pink datapoints) and the MEM approaches Kaiju and PRROMenade (blue datapoints) for simulated reads as substitution rate increases (x-axis). The linear trendline for the datapoints is shown in black with its respective linear equation. The red line denotes the average classification rate of the soil metagenomic samples considering both Kraken2, Kaiju and PRROMenade to estimate the substitution rate of these samples if we were to attempt to recreate them from simulated reads. Figure S2. Read alignment of 189 soil metagenomic samples to GO functional groups. Scatterplots to show the proportion of classified reads to each GO term for each of the 189 analysed soil samples after classification to the IFGP GO term sequence database using Kraken2 (read numbers normalized to a scale of 0-1 for each sample to allow comparison). The respective trendlines of closest fit for the datapoints are shown in red. Here we show classification summarized for specific GO functional groups as follows; (a) GO:0016209 for antioxidant activity, (b) GO:0045735 for nutrient reservoir activity, (c) GO:0005488 for binding, (d) GO:0098772 for molecular function regulation, (e) GO:0005198 for structural molecule activity, (f) GO:0003824 for catalytic activity, (g) GO:0005215 for transporter activity and (h) GO:00140110 for transcription regulator activity. Table S1. Classification of simulated sequencing reads (low substitution rate) by Kraken using three different sized GO term sequence databases (DNA based) and functional annotation. Table S2. Classification of 474,930,848 simulated sequencing reads (low, medium and high substitution rate) by Kraken, Kraken2 and the MEM approaches Kaiju and PRROMenade using the full IFGP GO term sequence database and functional annotation. Table S3. Classification of real soil metagenome sequencing reads by Kr [file 40168_2020_971_MOESM2_ESM.docx]

**Additional File 1: Supplementary Material**

**Figure S1. Estimation of substitution rate of soil metagenomic samples.** Scatterplot to show the classification rate (y-axis) of Kraken2 (pink datapoints) and the MEM approaches Kaiju and PRROMenade (blue datapoints) for simulated reads as substitution rate increases (x-axis)**.** The linear trendline for the datapoints is shown in black with its respective linear equation. The red line denotes the average classification rate of the soil metagenomic samples considering both Kraken2, Kaiju and PRROMenade to estimate the substitution rate of these samples if we were to attempt to recreate them from simulated reads.

**Figure S2. Read alignment of 189 soil metagenomic samples to GO functional groups.** Scatterplots to show the proportion of classified reads to each GO term for each of the 189 analyzed soil samples after classification to the IFGP GO term sequence database using Kraken2 (read numbers normalized to a scale of 0-1 for each sample to allow comparison). The respective trendlines of closest fit for the datapoints are shown in red. Here we show classification summarized for specific GO functional groups as follows; **(a)** GO:0016209 for antioxidant activity, **(b)** GO:0045735 for nutrient reservoir activity, **(c)** GO:0005488 for binding, **(d)** GO:0098772 for molecular function regulation, **(e)** GO:0005198 for structural molecule activity, **(f)** GO:0003824 for catalytic activity, **(g)** GO:0005215 for transporter activity and **(h)** GO:00140110 for transcription regulator activity.

**Table S1. Classification of simulated sequencing reads (low substitution rate) by Kraken using three different sized GO term sequence databases (DNA based) and functional annotation**.

| GO term database | Simulated Read number | Non-classified reads (un-mapped) | Classified Reads (%) | % Classified reads assigned to root LCF | % Classified reads assigned to correct GO term or related GO term | % Classified reads assigned to an incorrect GO term- |
| --- | --- | --- | --- | --- | --- | --- |
| Bordetella | 1,331,398 | 212,096 (15.93%) | 1,119,302 (84.07%) | 7.53 | 92.46 | 0.01 |
| Salmonella | 351,270,640 | 53,988,830 (15.37%) | 297,281,810 (84.63%) | 37.60 | 61.45 | 0.95 |
| IFGP complete GO | 474,930,848 | 70,286,855 (14.80%) | 404,643,993 (85.20%) | 14.20 | 84.10 | 1.70 |

**Table S2. Classification of 474,930,848 simulated sequencing reads (low, medium and high substitution rate) by Kraken, Kraken2 and the MEM approaches Kaiju and PRROMenade using the full IFGP GO term sequence database and functional annotation.**

| Software | Substitution Rate | Database format | Non-classified reads (un-mapped) | Classified Reads (%) | % Classified reads assigned to root LCF | % Classified reads assigned to correct GO term or related GO term | % Classified reads assigned to an incorrect GO term- |
| --- | --- | --- | --- | --- | --- | --- | --- |
| Kraken | 0.05 | DNA | 70,286,855 (14.80%) | 404,643,993 (85.20%) | 14.20 | 84.10 | 1.70 |
| Kraken2 | 0.05 | Amino Acid | 28,911,414  (6.09%) | 446,019,434  (93.91%) | 13.71 | 86.29 | 1.12 |
| MEM | 0.05 | Amino Acid | 70,577,941  (14.86%) | 404,352,907  (85.14%) | 14.61 | 84.71 | 0.68 |
| Kraken | 0.1 | DNA | 290,537,982  (61.17%) | 184,392,866  (38.83%) | 14.66 | 83.32 | 2.02 |
| Kraken2 | 0.1 | Amino Acid | 207,449,338  (43.68%) | 267,481,510  (56.32%) | 14.20 | 82.52 | 3.28 |
| MEM | 0.1 | Amino Acid | 148,138,444 (31.19%) | 326,792,404  (68.81%) | 14.86 | 83.32 | 1.83 |
| Kraken | 0.2 | DNA | 426,894,017  (97.47%) | 12,036,831  (2.53%) | 14.97 | 81.60 | 3.43 |
| Kraken2 | 0.2 | Amino Acid | 435,359,794  (91.67%) | 39,571,054  (8.33%) | 12.79 | 62.52 | 24.69 |
| MEM | 0.2 | Amino Acid | 426,293,852 (89.76%) | 48,636,996  (10.24%) | 14.27 | 75.69 | 10.04 |

**Table S3. Classification of real soil metagenome sequencing reads by Kraken, Kraken2 and the MEM approaches Kaiju and PRROMenade using the full IFGP GO term sequence database and functional annotation**.

| Software | Sample | Database format | Read number | Non-classified reads (un-mapped) | Classified Reads (%) | % Classified reads assigned to GO term |
| --- | --- | --- | --- | --- | --- | --- |
| Kraken | AV110 (Moist tropical forest) | DNA | 1,112,964 | 1,093,609 (98.26%) | 19,355 (1.74%) | 88.69 |
| Kraken | AV112 (Moist tropical forest) | DNA | 940,050 | 924,269 (98.32%) | 15,781 (1.68%) | 89.02 |
| Kraken | S234 (Boreal forest) | DNA | 1,137,027 | 1,118,337 (98.36%) | 18,690 (1.64%) | 88.74 |
| Kraken | S238 (Boreal forest) | DNA | 1,076,317 | 1,059,838 (98.47%) | 16,479 (1.53%) | 88.79 |
| Kraken2 | AV110 (Moist tropical forest) | Amino Acid | 1,112,964 | 709,428  (63.74%) | 403,536  (36.26%) | 92.54 |
| Kraken2 | AV112 (Moist tropical forest) | Amino Acid | 940,050 | 595,946  (63.40%) | 344,104  (36.60%) | 93.25 |
| Kraken2 | S234 (Boreal forest) | Amino Acid | 1,137,027 | 728,341  (64.06%) | 408,686  (35.94%) | 93.17 |
| Kraken2 | S238 (Boreal forest) | Amino Acid | 1,076,317 | 690,265  (64.13%) | 386,052  (35.87%) | 93.66 |
| MEM | AV110 (Moist tropical forest) | Amino Acid | 1,112,964 | 715,447 (64.28%) | 397,517 (35.72%) | 93.28 |
| MEM | AV112 (Moist tropical forest) | Amino Acid | 940,050 | 587,558 (62.50%) | 352,492 (37.50%) | 93.38 |
| MEM | S234 (Boreal forest) | Amino Acid | 1,137,027 | 734,390 (64.59%) | 402,637 (35.41%) | 93.06 |
| MEM | S238 (Boreal forest) | Amino Acid | 1,076,317 | 698,891 (64.93%) | 377,426 (35.07%) | 93.41 |
