# Supplementary material for: Re-purposing software for functional characterization of the microbiome
Source: Microbiome. 2021 Jan 9;9:4. doi: 10.1186/s40168-020-00971-1 (PMC7797099; doi:10.1186/s40168-020-00971-1)
Supplement: Supplementary file 6 — Additional file 5. A step by step description of how to use our GO functional hierarchy to build reference databases and perform classification for Kraken2 and Kaiju. [file 40168_2020_971_MOESM6_ESM.docx]

**Additional File 5: Step by step instructions of how to use our GO functional hierarchy to build a reference database for Kraken2/Kaiju**

**1. Introduction**

Here we detail the full GO hierarchy with a format and file structure ready to use for building a reference database with Kraken2 or Kaiju. For demonstration purposes, we then provide a small protein GO sequence database (200 sequences) that is a subset of the full GO sequence database used in the main manuscript. We give sample instructions for building reference databases from the GO sequence database plus GO hierarchy using either Kraken2 and Kaiju. If a different GO database is desired for usage downstream, the demonstration database here can be replaced as necessary.

**2. Building a reference database using the GO taxonomy (Kaiju: Additional File 6)**

1. Follow instructions for download and installation of Kaiju from official Kaiju user manual [available at: https://github.com/bioinformatics-centre/kaiju/blob/master/README.md]

Here we use version: Kaiju 1.7.2

1. Run the following command on Additional File 6 to unpack:

tar -xzf AdditionalFile6-Kaiju_GO_DB.tar.gz

1. Change directory into the newly created “AdditionalFile6-Kaiju_GO_DB” and stay in this directory for the following commands to run successfully.
2. To create custom Kaiju database as per Kaiju official instructions run:

kaiju-mkbwt -a protein SampleKaiju_GO_DB_protein_seqs.faa

kaiju-mkfmi SampleKaiju_GO_DB_protein_seqs.faa

1. For Kaiju classification, we replace the NCBI taxonomy file nodes.dmp with our GO hierarchy file of the same name nodes.dmp provided in the current working folder. To run classification, type the following (replacing files/arguments in bold with your own):

kaiju -t nodes.dmp -f SampleKaiju_GO_DB_protein_seqs.faa.fmi **-I** **Your_Reads.fq.gz** **-j Your_Reads2.fq.gz** **-z 100 -a mem -o** **Test_output**

**3. Building a reference database using the GO taxonomy (Kraken2: Additional File 7 and 8)**

1. Follow instructions for download and installation of Kraken2 from official Kraken2 user manual [available at: https://github.com/DerrickWood/kraken2/wiki/Manual]

Here we use version: Kraken version 2.0.8-beta

1. Copy Additional File 7 and Additional File 8 to the same location. Stay at this location in your file system until directed otherwise.
2. Run the following command on Additional File 7 to unpack:

tar -xzf AdditionalFile7-Kraken2_GO_DB.tar.gz

1. Run the following command to add our sample GO sequence database to the pre-existing GO hierarchy that is held within the newly created “AdditionalFile7-Kraken2_GO_DB” directory from step (c) (replacing arguments in bold with your own):

kraken2-build --add-to-library AdditionalFile8-Sample_GO_DB_protein_seqs.faa --db AdditionalFile7-Kraken2_GO_DB **--no-masking**

1. To create custom Kraken2 database run:

kraken2-build --build -db AdditionalFile7-Kraken2_GO_DB --protein

1. The database is now ready for use. For example, for Kraken2 classification, we can now type the following as per Kraken2 official instructions (replacing files/arguments in bold with your own):

kraken2 --db AdditionalFile7-Kraken2_GO_DB --paired **Your_Reads.fq.gz** **Your_Reads2.fq.gz** **--gzip-compressed** **>** **Test_output**
